# Dissociation Between Symptom Burden and Structural Pathology in Patients Undergoing Upper Gastrointestinal Endoscopy: A Retrospective Single-Center Study Using Latent Class Analysis

**DOI:** 10.3390/medicina62102035

**Published:** 2026-10-21

**Authors:** Linda Grantiņa, Evija Nagle, Maksims Zolovs, Dace Dosone, Olga Sjomina, Danute Ražuka-Ebela, Eva Petrošina, Jeļena Perevožčikova, Alina Zolova

**Affiliations:** 1 Statistics Unit, Riga Stradiņš University, Dzirciema Street 16, LV-1007 Riga, Latvia; 2 Digestive Diseases Centre GASTRO, Gaiļezera Street 1, LV-1079 Riga, Latvia; 3 Institute of Life Sciences and Technology, Daugavpils University, Vienības Street 13, LV-5401 Daugavpils, Latvia; 4 Institute of Clinical and Preventive Medicine, University of Latvia, Gaiļezera Street 1, LV-1079 Riga, Latvia; 5 Department of Rehabilitation, Riga Stradiņš University, Anniņmuižas Bulvāris 26a, LV-1067 Riga, Latvia; 6 Institute of Food and Environmental Hygiene, Latvia University of Life Sciences and Technologies, K.Helmaņa Street 8, LV-3004 Jelgava, Latvia

**Keywords:** upper gastrointestinal endoscopy, latent class analysis, symptom burden, clinical features, psychosocial factors, histopathology, risk stratification

## Abstract

*Background and Objectives*: Upper gastrointestinal symptoms often correspond poorly with endoscopic and histological pathology. We aimed to identify lifestyle and psychosocial phenotypes and examine their clinical features, objective findings, and symptom–pathology concordance. *Materials and Methods*: This retrospective single-center study included adults undergoing outpatient upper gastrointestinal endoscopy at a tertiary gastroenterology center in Latvia between 1 May and 31 December 2025. Latent class analysis was applied to questionnaire-derived lifestyle, behavioral, anthropometric, and psychosocial indicators. Classes were subsequently characterized using clinical features, endoscopic findings, and histology. Additional analyses assessed potential confounding by age and sex and exploratory predictive discrimination. *Results*: The cohort included 2192 patients, of whom 1755 had histology. A three-class solution had the lowest mean Bayesian Information Criterion, with substantial agreement across 20 imputed datasets. Class 1 (46.9%) combined high psychosocial burden with comparatively low substance exposure. Class 2 (12.0%) combined greater substance exposure with high psychosocial burden and the highest anxiety prevalence. Class 3 (41.0%) exhibited lower psychosocial burden, less prolonged sitting, fewer patients with short sleep duration, and the highest combined prevalence of overweight and obesity. The clinical feature composite showed limited diagnostic performance, and correlations between clinical feature burden and pathology burdens were negligible (Spearman’s ρ = −0.012 to 0.026; all *p* > 0.05). Clinical feature-dominant discordance was most frequent in Class 2 (25.5%), whereas pathology-dominant discordance was most frequent in Class 3 (29.7%). Eleven clinical-feature associations remained significant after age and sex adjustment and false discovery rate correction; no overall associations with individual endoscopic or histological findings met this threshold. Exploratory ROC analyses showed limited discrimination. *Conclusions*: The phenotypes demonstrated distinct patterns of symptom–pathology dissociation. Lifestyle-based phenotyping provides a framework for characterizing this heterogeneity, while its clinical application requires validation in independent populations.

## 1. Introduction

Upper gastrointestinal (UGI) symptoms are among the most common reasons for seeking medical care in gastroenterology and represent a major driver of referral for diagnostic upper gastrointestinal endoscopy. Symptoms such as epigastric pain, heartburn, and dyspeptic symptoms are frequent in clinical practice. Because these symptoms often determine whether patients are referred for endoscopic evaluation, symptom assessment plays a central role in real-world clinical decision-making. This makes the relationship between symptom burden and endoscopic pathology clinically important [1,2,3]. UGI diseases constitute a substantial public health burden both globally and across Europe [4].

However, a major limitation of symptom-based decision-making is that symptoms do not necessarily correspond to structural disease. Many patients with severe or persistent UGI symptoms have normal endoscopic findings or only minor abnormalities, whereas some patients with clinically relevant pathology report minimal, atypical, or nonspecific symptoms. This mismatch suggests that symptom burden and structural pathology should not be treated as interchangeable constructs [5,6,7].

In addition, symptom generation and symptom perception may be influenced by psychosocial and behavioral factors independent of structural pathology. Emerging evidence from disorders of gut–brain interaction suggests that stress, anxiety, and related psychosocial mechanisms may amplify gastrointestinal symptom burden even in the absence of clinically significant endoscopic abnormalities [8]. These observations further support the concept that patient-reported symptoms and objective pathology may represent partially dissociated clinical dimensions.

Despite the central role of symptoms in clinical triage, the diagnostic yield of upper gastrointestinal endoscopy remains relatively low. Current guidelines recommend referral for endoscopy primarily based on alarm symptoms and relevant risk factors. However, many patients presenting with UGI symptoms do not have clinically significant pathology. A large-scale analysis conducted by the British Society of Gastroenterology, based on 382,370 diagnostic gastroscopies, found that 89.9% of examinations showed normal findings or only minor pathology [9]. This discrepancy highlights a critical inefficiency in current diagnostic pathways and underscores the need for improved risk stratification approaches.

In clinical practice, patient selection for endoscopy is largely guided by the presence of alarm symptoms. However, the predictive value of these symptoms alone remains limited. Evidence suggests that a considerable proportion of patients with clinically significant pathology may be present without alarm symptoms. In a multicenter study involving more than 21,000 patients, clinically significant endoscopic findings were identified in only 9.5% of individuals presenting with alarm symptoms, with an upper gastrointestinal cancer prevalence of 1.7% [10]. Similarly, a study conducted in Northeast Brazil found no statistically significant association between reported symptoms and endoscopic outcomes [11]. These results indicate that reliance solely on symptom-based risk stratification may lead to both overutilization of endoscopy and missed identification of high-risk patients.

Given these limitations, lifestyle-related factors have emerged as important determinants of gastrointestinal disease. Obesity, sedentary behavior, smoking, alcohol use, and physical inactivity have all been associated with increased risk of upper gastrointestinal pathology and symptom burden [12,13,14]. However, existing research predominantly evaluates these factors at the population level and rarely integrates them into clinical decision-making frameworks for symptomatic patients.

A critical gap in the current literature is the absence of integrative approaches that consider both clinical symptoms and modifiable lifestyle factors within a unified analytical framework. Most studies have examined these domains separately, thereby limiting the accurate identification of clinically meaningful patient subgroups in real-world settings [7,8,15].

Person-centered analytical approaches, such as latent class analysis (LCA), offer a robust alternative to traditional variable-centered methods. These approaches enable the identification of distinct patient phenotypes based on shared patterns of clinical, behavioral, and lifestyle characteristics. Such approaches could support more targeted and efficient diagnostic strategies [16,17].

The present study was designed to characterize phenotypic and symptom–pathology patterns within a defined tertiary gastroenterology setting rather than to derive population-representative estimates or imply direct generalizability across healthcare systems. At the same time, the single-center design provided a comparatively consistent data-generating environment, with a common pre-endoscopy questionnaire, shared institutional workflows, and histological assessment within a single pathology laboratory, thereby enhancing internal consistency and limiting between-center heterogeneity in data capture and diagnostic reporting.

Within this context, we aimed to identify clinically meaningful patient phenotypes among individuals undergoing upper gastrointestinal endoscopy by applying latent class analysis to lifestyle, behavioral, and psychosocial characteristics, and subsequently to evaluate how the identified phenotypes differed in symptom burden, endoscopic findings, histological pathology, and symptom–pathology concordance patterns.

## 2. Materials and Methods

### 2.1. Study Design and Setting

This retrospective observational study aimed to identify latent patient phenotypes among individuals undergoing upper gastrointestinal endoscopy. Latent phenotypes were identified based on patterns of lifestyle, behavioral, and psychosocial characteristics. Phenotypes were subsequently compared with respect to gastrointestinal symptoms, endoscopic findings, and histological outcomes. The study was conducted at Digestive Diseases Centre GASTRO, a tertiary gastroenterology center in Latvia. Data from standardized digital questionnaires completed before endoscopy were linked to endoscopic and histological findings. Patients completed the questionnaire before the procedure as part of the routine clinical workflow. Endoscopic examinations, including biopsy sampling at the endoscopist’s discretion, were performed as part of routine clinical care, and histological analyses were performed in accordance with routine diagnostic procedures. The study period extended from 1 May 2025 to 31 December 2025.

### 2.2. Study Population

The study population comprised adult outpatients who underwent upper gastrointestinal endoscopy at a single center during the study period and had corresponding questionnaire data available. Patients underwent upper gastrointestinal endoscopy in the outpatient setting and were referred predominantly by family physicians. A total of 2192 individuals were included in the analysis. To avoid repeated inclusion of the same individual, only the first upper endoscopy performed during the study period was considered for patients who underwent more than one examination. Patients were eligible if both questionnaire responses and endoscopic findings were available. Histological data from biopsy specimens were available for a subset of 1755 patients and were included in analyses involving histological outcomes. No additional exclusion criteria were applied beyond data availability and consistency.

### 2.3. Data Collection

Data were obtained from a standardized digital questionnaire completed prior to endoscopy. The questionnaire included demographic, anthropometric, clinical, lifestyle, and psychosocial variables. Endoscopic findings were obtained from clinical reports generated during the procedure. Biopsy sampling was performed at the endoscopist’s discretion as part of routine clinical care, and the corresponding histological findings were obtained from pathology reports. A prespecified subset of questionnaire variables was selected for latent class analysis, whereas symptom, endoscopic, and histological variables were used for post hoc profiling of the derived classes.

ICD-10 referral diagnoses were obtained from the referring physicians’ referrals, with one primary diagnosis recorded per patient. These diagnoses were grouped into mutually exclusive clinical categories for descriptive reporting.

### 2.4. Variables

#### 2.4.1. Variables Included in Latent Class Analysis

Nicotine and alcohol exposure indicators were derived from questionnaire responses on use status, frequency, and, for alcohol, consumption quantity. Recreational drug use was coded as positive when either lifetime or current use was reported. Alcohol exposure was represented by a single four-category indicator combining alcohol-use status and current consumption intensity: never use, former use, current low consumption, and current moderate-to-high consumption. Lifestyle and psychosocial variables were categorized using clinically meaningful thresholds. Sedentary behavior, sleep duration, perceived stress, and BMI were categorized into ordered groups, whereas physical activity, sleep medication use, increased stress, anxiety, and occupational risk exposure were treated as binary indicators. BMI categories were based on standard clinical thresholds. Detailed operational definitions and category coding for all variables are presented in Table 1.

#### 2.4.2. Variables Used for Post Hoc Profiling

Variables not included in the LCA model were used to characterize differences between latent classes. Clinical variables included gastrointestinal and extraintestinal symptoms and signs, such as abdominal pain, bloating, fatigue, reflux-related symptoms, belching, nausea, sweating, appetite changes, anemia, unexplained weight loss, dysphagia-related symptoms, and vomiting. These variables were coded as binary indicators. The study-specific clinical feature composite comprised abdominal pain, fatigue, excessive sweating, anemia, unexplained weight loss, pain on swallowing, swallowing difficulties, vomiting, and hematemesis, and was positive when at least one feature was reported. Clinical feature burden was calculated as the number of these nine selected features reported by each patient, ranging from 0 to 9.

Endoscopic variables included absence of pathology, cardia insufficiency, gastropathy, grade A/B and grade C/D reflux esophagitis, hiatal hernia, gastric erosions, endoscopic atrophy, bile reflux, post-gastric resection changes, gastric and duodenal ulcers, esophageal varices, Barrett’s esophagus, achalasia, gastric and esophageal tumors, esophageal stricture, pyloric stenosis, and Mallory–Weiss tear. Each finding was coded as a binary indicator.

Histological variables extracted from biopsy reports included *Helicobacter pylori* infection, low-grade or high-grade dysplasia of the stomach or esophagus, including Barrett’s-associated dysplasia, gastritis, gastropathy, intestinal metaplasia, duodenopathy, neoplasia, as well as stages of gastric atrophy and intestinal metaplasia according to the Operative Link on Gastritis Assessment (OLGA) and Operative Link on Gastric Intestinal Metaplasia Assessment (OLGIM) systems, respectively. Neoplasia comprised histologically confirmed carcinoma, neuroendocrine tumors, or metastatic melanoma. For analysis, patients were assigned to combined OLGA/OLGIM categories—stage 0, stages I–II, or stages III–IV—according to the higher recorded OLGA or OLGIM stage. Duodenopathy could only be interpreted among patients in whom duodenal biopsies were obtained; however, standardized information on biopsy site was not available. All histological variables were coded as binary indicators.

Three binary outcomes were defined for this study. Histological yield was considered positive when at least one of the following was identified: histologically confirmed neoplasia, low-grade or high-grade dysplasia, *Helicobacter pylori* infection, or advanced gastric atrophy or intestinal metaplasia within the combined OLGA/OLGIM stage III-IV category, based on the higher recorded OLGA or OLGIM stage. Endoscopic yield was considered positive when at least one of the following was identified: grade C/D reflux esophagitis, gastric ulcer, duodenal ulcer, esophageal varices, Schatzki ring, Barrett’s esophagus, achalasia, gastric tumor, esophageal stricture, esophageal tumor, pyloric stenosis, or Mallory-Weiss tear. High-yield gastroscopy was defined as a positive endoscopic yield, a positive histological yield, or both.

The yield outcomes were study-specific composites of selected findings rather than indicators of any endoscopic or histological abnormality. Isolated gastritis, gastropathy, duodenopathy, gastric erosions, or grade A/B reflux esophagitis did not qualify as positive yield unless another qualifying finding was present. Similarly, intestinal metaplasia or combined OLGA/OLGIM stages I–II alone did not meet the histological-yield definition. The composites included selected benign structural or acid-related findings, *H. pylori* infection, premalignant changes, and histologically confirmed neoplasia. Inclusion did not imply equivalent severity or urgency of management, and high-yield gastroscopy was not a malignancy-specific outcome.

Endoscopic burden was calculated as the number of findings included in the endoscopic-yield definition and histological burden—as the number of findings included in the histological-yield definition. Combined pathology burden was the sum of endoscopic and histological burdens.

### 2.5. Statistical Analysis

Missing data in LCA indicator variables were addressed using multiple imputations by chained equations under the assumption of missing at random. Twenty imputed datasets were generated, and only variables included in the LCA model were entered into the imputation procedure.

Latent class analysis was performed separately within each imputed dataset using maximum likelihood estimation. Models with one to six classes were evaluated, and model selection was based primarily on the Bayesian Information Criterion (BIC) across the 20 imputed datasets, with additional consideration of entropy, class size distribution, and consistency across imputations.

After selecting the number of classes, the fitted model with the lowest BIC among the 20 imputed datasets was chosen as the representative model for post hoc profiling. Patients were assigned to latent classes according to their highest posterior membership probability in this model. Behavioral, lifestyle, and psychosocial characteristics were summarized using the completed indicator data from the representative imputed dataset. Demographic characteristics and clinical, endoscopic, and histological findings were summarized using observed data, with percentages calculated from the available observations for each variable within each class.

Latent classes were subsequently characterized according to symptom, endoscopic, and histological variables. Categorical clinical findings were compared across classes using Pearson’s chi-square test. For histological findings, Fisher’s exact test was used when any expected cell count was below five. Continuous or count variables were compared using the Kruskal–Wallis test. *p*-values for comparisons of clinical findings were adjusted using the Benjamini–Hochberg false discovery rate procedure, separately within the symptom, endoscopic, and histological domains.

The diagnostic performance of the clinical feature composite for endoscopic yield, histological yield, and high-yield gastroscopy was evaluated using sensitivity, specificity, positive predictive value, and negative predictive value, with exact binomial 95% confidence intervals.

Associations between clinical feature burden and endoscopic, histological, and combined pathology burdens were assessed using Spearman’s rank correlation. Each analysis included patients with complete data for the relevant burden measures. Endoscopic-burden analyses used the full endoscopy cohort, whereas histological- and combined-burden analyses were restricted to patients with histological data.

Clinical feature-pathology concordance was assessed among patients with histological data by combining clinical feature burden with high-yield gastroscopy status. Low clinical feature burden was defined as 0–1 features and high burden as ≥2 features. Four profiles were defined: concordant low (low burden and negative yield), concordant high (high burden and positive yield), clinical feature-dominant discordance (high burden and negative yield), and pathology-dominant discordance (low burden and positive yield). Profile distributions were compared across latent classes using Pearson’s chi-square test, with association strength quantified using Cramér’s V.

To assess potential selection related to histology availability, the behavioral, lifestyle, and psychosocial characteristics were compared between patients with and without histological data using observed responses before imputation. Comparisons used Pearson’s chi-square tests, with Cramér’s V as the effect-size measure. *p*-values were adjusted using the Benjamini–Hochberg procedure.

Potential confounding by age and sex was examined using separate binary logistic regression models for each clinical feature, endoscopic finding, and histological finding. Unadjusted models included latent class membership as a categorical predictor; adjusted models additionally included continuous age and sex. The existing latent class assignments were retained, and both models used the same eligible patients for each finding. Histological analyses were restricted to patients with available histology. Mean bias-reduced estimation was used to accommodate sparse outcomes and separation. Overall associations with class membership were assessed using joint two-degree-of-freedom Wald tests. Benjamini–Hochberg correction was applied separately within each domain and separately to the unadjusted and adjusted tests.

Exploratory prediction analyses were performed separately for endoscopic and histological yield using outcome-stratified training/test splits of approximately 70%/30%. Histological-yield analyses were restricted to patients with available histology. Imputation and three-class LCA models were fitted using training data only, without including the pathology outcome in either procedure. Three logistic regression models were evaluated: the clinical feature composite alone, latent class membership alone, and their combination. Latent class membership was entered as a categorical predictor, and mean bias-reduced estimation was used. Within each outcome, models were evaluated using the same held-out test patients, with predicted probabilities averaged across the 20 imputations. Discrimination was assessed using receiver operating characteristic curves and AUCs with 95% DeLong confidence intervals. AUC differences were assessed using paired DeLong tests, with Holm correction across six comparisons within each outcome, including comparisons involving additional age- and sex-adjusted models.

Consistency of latent class assignments across the 20 imputed datasets was evaluated using pairwise adjusted Rand indices. Statistical significance was defined as *p* < 0.05; where multiple-comparison correction was applied, interpretation was based on the corresponding adjusted *p*-values. All analyses were performed using R (R Foundation for Statistical Computing, Vienna, Austria).

## 3. Results

### 3.1. Study Population Characteristics

A total of 2192 patients undergoing upper gastrointestinal endoscopy were included in the analysis (Table 2). The mean age was 50.8 ± 14.9 years, and 66.4% of participants were female. The mean body mass index (BMI) was 26.6 ± 5.3 kg/m^2^; 33.1% of participants were overweight, and 23.7% were classified as obese. Most participants were highly educated (59.5%) and employed (62.8%).

Referral diagnoses were available for all 2192 patients. The most frequent categories were gastritis and duodenitis (25.9%), gastroesophageal reflux disease and esophagitis (24.7%), and observation for suspected malignancy (21.9%). Other referral categories included peptic ulcer disease, neoplasms, functional dyspepsia, intestinal disorders, and anemia or iron deficiency. The distribution of recorded referral diagnoses is presented in Table 3.

### 3.2. Diagnostic Performance of the Clinical Feature Composite

Before evaluating latent phenotypes, we examined the diagnostic performance of a clinical feature composite for identifying clinically meaningful pathology (Table 4). The composite was defined as the presence of at least one of the following: abdominal pain, fatigue, excessive sweating, anemia, unexplained weight loss, pain on swallowing, swallowing difficulties, vomiting, or hematemesis.

The clinical feature composite showed limited diagnostic performance across the three outcome definitions (Table 4). Sensitivity was 0.48 for endoscopic yield and 0.44 for both histological yield and high-yield gastroscopy, while specificity ranged from 0.55 to 0.56. Positive predictive values were 0.05, 0.28, and 0.30, respectively; corresponding negative predictive values were 0.96, 0.72, and 0.69. Although the negative predictive value was higher for endoscopic yield, this should be interpreted in the context of its low prevalence. For high-yield gastroscopy, the limited sensitivity and negative predictive value indicated that absence of the selected clinical features did not reliably exclude objective pathology.

Spearman correlation analysis demonstrated no meaningful association between clinical feature burden and objectively detected pathology burden. Across endoscopic, histological, and combined pathology burden scores, correlation coefficients were close to zero and effect sizes were negligible, indicating that patients with a higher number of reported clinical features did not show a corresponding increase in endoscopic or histological abnormalities. These findings further support the presence of a dissociation between patient-reported feature burden and objective structural or histological pathology in this cohort (Table 5).

To further assess clinical feature-pathology alignment, patients were categorized into concordant and discordant profiles (Table 6). Concordant low findings were the most frequent profile (53.4%), followed by pathology-dominant discordance (23.8%), clinical feature-dominant discordance (16.2%), and concordant high findings (6.6%). Thus, discordance occurred in both directions, with objective pathology despite low clinical feature burden occurring more frequently than high clinical feature burden without objective pathology.

### 3.3. Latent Class Model Selection

Latent class models with one to six classes were evaluated using Bayesian Information Criterion (BIC), entropy, and class size distribution (Table 7). The three-class solution demonstrated the lowest mean BIC (37,237.2 ± 39.1) and was favored in all 20 imputed datasets. Its mean entropy was 0.655 ± 0.005, and the smallest assigned class represented an average of 13.4% of participants across imputations. The four-class solution had a higher mean BIC and lower mean smallest class, whereas the five- and six-class solutions included classes comprising less than 10% of participants. Class assignments were largely consistent across imputed datasets, with a mean pairwise adjusted Rand index of 0.863 ± 0.028. Accordingly, the three-class solution was selected for subsequent analyses.

### 3.4. Characterization of Latent Classes

The representative three-class solution assigned 1029 participants (46.9%) to Class 1, 264 (12.0%) to Class 2, and 899 (41.0%) to Class 3. Demographic characteristics across classes are presented in Table 8, and behavioral, lifestyle, and psychosocial characteristics are presented in Table 9.

Demographic characteristics differed across classes (Table 8). Class 2 had the lowest mean age (41.6 ± 13.2 years), followed by Class 1 (48.9 ± 14.4 years) and Class 3 (55.6 ± 14.1 years). Women comprised 76.6% of Class 1, compared with 54.5% of Class 2 and 58.2% of Class 3. Higher education was more frequent in Class 1 (66.4%) than in Classes 2 (57.6%) and 3 (52.2%). Employment was less frequent in Class 3 (56.8%) than in Classes 1 (67.2%) and 2 (66.3%). Overall differences in age, sex, education, and employment were statistically significant (all *p* < 0.001).

**Table 8:** Demographic characteristics across latent classes.

Characteristic	Class 1 (n = 1029)	Class 2 (n = 264)	Class 3 (n = 899)	*p*-Value	Effect Size
Age, years, mean ± SD	48.9 ± 14.4 ^a^	41.6 ± 13.2 ^b^	55.6 ± 14.1 ^c^	<0.001	0.096
Sex, n (%)				<0.001	0.204
Female	788 (76.6) ^a^	144 (54.5) ^b^	523 (58.2) ^b^		
Male	241 (23.4) ^a^	120 (45.5) ^b^	376 (41.8) ^b^		
Education, n (%)				<0.001	0.120
Available data, n	1014	262	881		
Higher education	673 (66.4) ^a^	151 (57.6) ^b^	460 (52.2) ^b^		
Primary education	6 (0.6) ^b^	10 (3.8) ^a^	13 (1.5) ^b^		
Vocational/technical education	168 (16.6) ^b^	42 (16.0) ^b^	227 (25.8) ^a^		
General secondary education	102 (10.1) ^a^	41 (15.6) ^b^	115 (13.1) ^ab^		
Other/prefer not to disclose	65 (6.4) ^a^	18 (6.9) ^a^	66 (7.5) ^a^		
Employment, n (%)				<0.001	0.149
Available data, n	1009	261	886		
Employed	678 (67.2) ^b^	173 (66.3) ^b^	503 (56.8) ^a^		
Unemployed	24 (2.4) ^b^	19 (7.3) ^a^	21 (2.4) ^b^		
Not working due to disability	19 (1.9) ^a^	6 (2.3) ^a^	22 (2.5) ^a^		
Homemaker	25 (2.5) ^a^	7 (2.7) ^a^	20 (2.3) ^a^		
Retired, not working	116 (11.5) ^a^	17 (6.5) ^b^	202 (22.8) ^c^		
Student, not working	23 (2.3) ^b^	9 (3.4) ^b^	5 (0.6) ^a^		
Other/prefer not to disclose	124 (12.3)^a^	30 (11.5) ^a^	113 (12.8) ^a^		

Note. Values are presented as mean ± standard deviation or number (percentage). Percentages use the available observations within each class. “Other” and “prefer not to disclose” responses were combined; missing responses were excluded. Overall comparisons used the Kruskal–Wallis test for age, Pearson’s chi-square test for sex, and Fisher’s exact test with Monte Carlo estimation (100,000 simulations) for education and employment. Effect sizes are eta-squared based on the Kruskal–Wallis H statistic (*η*^2^*H*) for age and Cramér’s V for categorical variables. Superscript letters indicate pairwise Wilcoxon tests for age and Fisher’s exact tests for categorical proportions, with Holm correction. Within each row, values sharing no letter differ significantly at adjusted *p* < 0.05. Age tests compare distributions. SD, standard deviation.

**Table 9:** Behavioral, lifestyle, and psychosocial characteristics across latent classes.

Characteristic	Class 1 (n = 1029)	Class 2 (n = 264)	Class 3 (n = 899)	*p*-Value	Cramér’s V
Nicotine current use, n (%)				<0.001	0.335
Frequent	107 (10.4) ^a^	164 (62.1) ^b^	171 (19.0) ^c^		
Occasional	14 (1.4) ^b^	33 (12.5) ^a^	13 (1.4) ^b^		
None in past 12 months	908 (88.2) ^a^	67 (25.4) ^b^	715 (79.5) ^c^		
Alcohol exposure, n (%)				<0.001	0.388
Never	809 (78.6) ^a^	6 (2.3) ^b^	661 (73.5) ^c^		
Former	76 (7.4) ^a^	153 (58.0) ^b^	136 (15.1) ^c^		
Current—low consumption	114 (11.1) ^a^	53 (20.1) ^b^	62 (6.9) ^c^		
Current—moderate/high consumption	30 (2.9) ^b^	52 (19.7) ^a^	40 (4.4) ^b^		
Any recreational drug use, n (%)				<0.001	0.402
Yes	0 (0.0) ^b^	51 (19.3) ^a^	3 (0.3) ^b^		
Sedentary behavior, n (%)				<0.001	0.131
≤4 h/day	251 (24.4) ^b^	68 (25.8) ^b^	363 (40.4) ^a^		
>4 and ≤8 h/day	544 (52.9) ^a^	123 (46.6) ^ab^	408 (45.4) ^b^		
>8 h/day	234 (22.7) ^b^	73 (27.7) ^b^	128 (14.2) ^a^		
Regular physical activity, n (%)				0.249	0.036
Yes	448 (43.5) ^a^	117 (44.3) ^a^	361 (40.2) ^a^		
Sleep duration, n (%)				<0.001	0.089
≤6 h/night	289 (28.1) ^b^	68 (25.8) ^b^	164 (18.2) ^a^		
>6 and ≤8 h/night	696 (67.6) ^b^	172 (65.2) ^b^	679 (75.5) ^a^		
>8 h/night	44 (4.3) ^a^	24 (9.1) ^b^	56 (6.2) ^ab^		
Sleep medication use, n (%)				<0.001	0.117
Yes	236 (22.9) ^b^	68 (25.8) ^b^	128 (14.2) ^a^		
Perceived stress level, n (%)				<0.001	0.557
Low	0 (0.0) ^b^	0 (0.0) ^b^	363 (40.4) ^a^		
Moderate	236 (22.9) ^b^	74 (28.0) ^b^	530 (59.0) ^a^		
High	793 (77.1) ^b^	190 (72.0) ^b^	6 (0.7) ^a^		
Increased stress, n (%)				<0.001	0.632
Yes	667 (64.8) ^b^	183 (69.3) ^b^	26 (2.9) ^a^		
Anxiety, n (%)				<0.001	0.406
Yes	392 (38.1) ^a^	152 (57.6) ^b^	65 (7.2) ^c^		
Work environment risks, n (%)				<0.001	0.102
Yes	218 (21.2) ^a^	74 (28.0) ^b^	140 (15.6) ^c^		
BMI category, n (%)				<0.001	0.122
Underweight	50 (4.9) ^b^	9 (3.4) ^b^	6 (0.7) ^a^		
Normal weight	448 (43.5) ^b^	118 (44.7) ^b^	315 (35.0) ^a^		
Overweight	292 (28.4) ^a^	98 (37.1) ^b^	336 (37.4) ^b^		
Obesity	239 (23.2) ^b^	39 (14.8) ^a^	242 (26.9) ^b^		

Note. Values are n (%). Percentages are calculated within each latent class. Overall comparisons used Pearson’s chi-square tests, with effect sizes reported as Cramér’s V. Overall *p*-values are unadjusted. Superscript letters indicate pairwise comparisons of category-specific proportions using Fisher’s exact tests with Holm correction across the three class comparisons within each category. Within the same row, values sharing no letter differ significantly at adjusted *p* < 0.05. BMI, body mass index.

As an additional assessment of potential biopsy-selection bias, the behavioral, lifestyle, and psychosocial characteristics presented in Table 9 were compared between patients with and without histological data. After Benjamini–Hochberg correction, statistically significant differences were observed only for recreational drug use (1.9% versus 4.8%; adjusted *p* = 0.002; Cramér’s V = 0.075) and BMI category distribution (adjusted *p* < 0.001; Cramér’s V = 0.102). These associations were of limited magnitude: the effect size for recreational drug use was below the conventional small-effect benchmark of 0.10, whereas that for BMI distribution corresponded to a small effect. No statistically significant differences were detected for the remaining characteristics.

Class 1 was characterized by high psychosocial burden with comparatively low substance exposure. High perceived stress and increased stress were reported by 77.1% and 64.8%, respectively, while anxiety was reported by 38.1%. Most participants reported never using alcohol (78.6%) and no nicotine use in the preceding 12 months (88.2%); no recreational drug use was recorded in the completed dataset. Sitting for >4 to ≤8 h/day was the most common sedentary category (52.9%). Short sleep duration (≤6 h/night; 28.1%) and sleep medication use (22.9%) were more frequent than in Class 3.

Class 2 combined greater substance exposure with high psychosocial burden. Frequent nicotine use (62.1%) and recreational drug use (19.3%) were most prevalent in this class. Former alcohol use was the largest alcohol category (58.0%), while current low and moderate/high consumption accounted for 20.1% and 19.7%, respectively. High perceived stress (72.0%) and increased stress (69.3%) were common, and anxiety prevalence was highest in this class (57.6%). Participants also reported the highest occupational risk exposure (28.0%). Short sleep (25.8%) was more frequent than in Class 3 and sleep medication use (25.8%) was the highest among all 3 classes; however this class had the largest proportion reporting >8 h of sleep per night (9.1%).

Class 3 was distinguished by lower psychosocial burden: perceived stress was predominantly low or moderate, while increased stress and anxiety were reported by 2.9% and 7.2%, respectively. Compared with Classes 1 and 2, more participants reported sitting ≤ 4 h/day (40.4%), and fewer reported sitting > 8 h/day (14.2%). Sleep duration was more commonly >6 to ≤8 h/night (75.5%), with fewer patients sleeping 6 or less hours per night (18.2%) and less sleep medication use (14.2%). Occupational risk exposure was lowest (15.6%). Nevertheless, this class had the highest combined prevalence of overweight and obesity (64.3%), indicating that lower psychosocial burden did not uniformly coincide with lower exposure to other health-related risk factors. Regular physical activity did not differ significantly across the three classes (*p* = 0.249; V = 0.036).

Psychosocial indicators showed the strongest separation across the assigned classes, particularly increased stress (Cramér’s V = 0.632), perceived stress level (V = 0.557), and anxiety (V = 0.406). Substance exposure also differed across classes, including recreational drug use (V = 0.402), alcohol exposure (V = 0.388), and nicotine use (V = 0.335). Differences in BMI, sedentary behavior, sleep duration, and sleep medication use were smaller.

### 3.5. Clinical Feature Profiles Across Latent Classes

**Figure 1:**
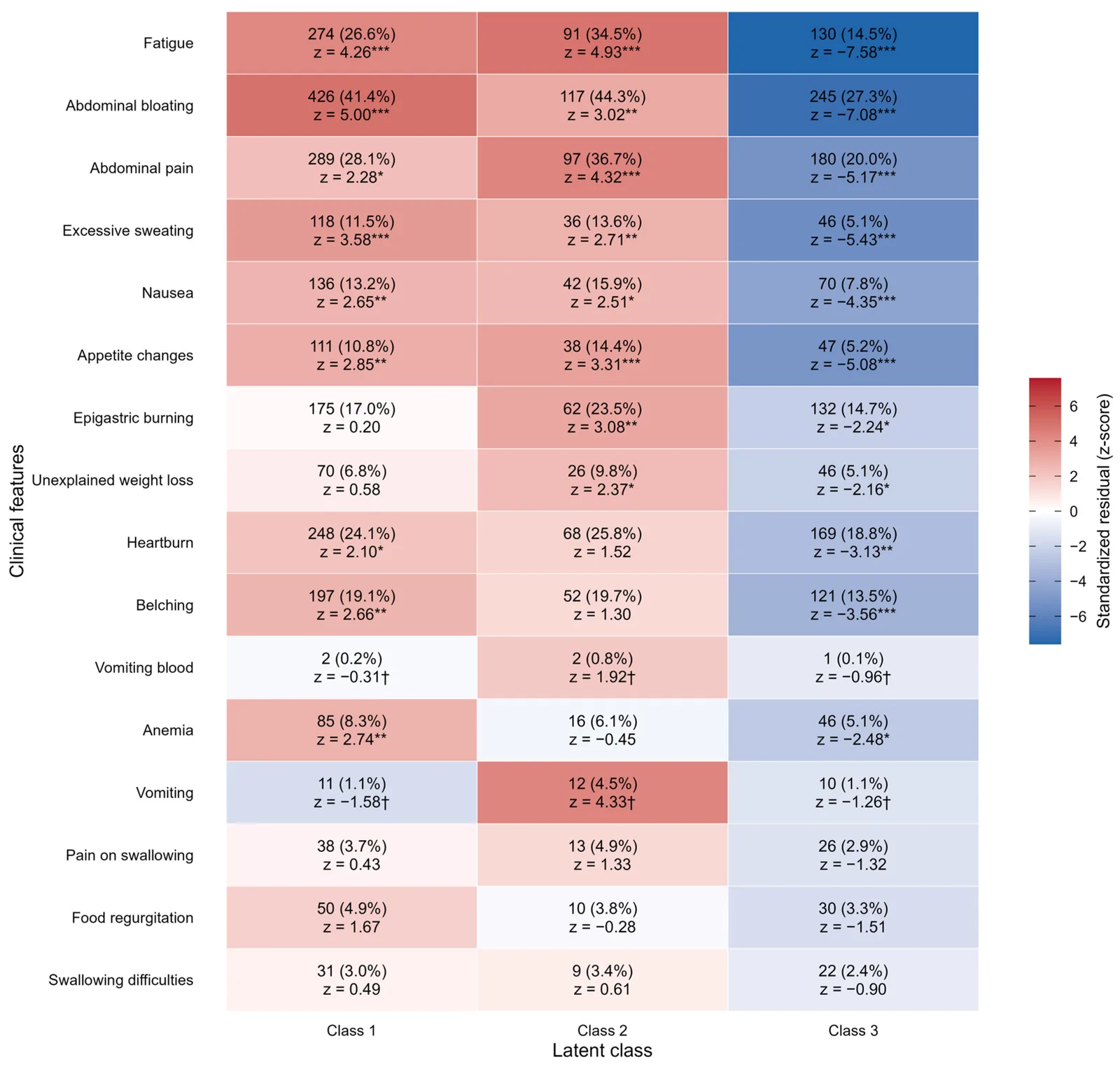
Clinical feature profiles across latent classes, presented as patient counts, within-class percentages, and standardized residuals. Each cell shows the number and within-class percentage of patients reporting the clinical feature, followed by its standardized residual (z). Red indicates more patients with the feature than expected under independence, and blue indicates fewer. One, two, and three asterisks denote exploratory thresholds of |z| > 1.96, > 2.58, and > 3.29, respectively. A dagger (†) identifies findings with sparse expected cell counts; their residuals are descriptive, and asterisks are omitted.

Clinical feature profiles varied across latent classes in their observed prevalences and relative distributions (Figure 1).

Class 2 had the highest observed prevalence of abdominal bloating (44.3%), abdominal pain (36.7%), fatigue (34.5%), nausea (15.9%), appetite changes (14.4%), and excessive sweating (13.6%). Class 1 showed intermediate prevalences of these features, whereas Class 3 consistently had the lowest prevalences: 27.3%, 20.0%, 14.5%, 7.8%, 5.2%, and 5.1%, respectively. Overall differences across classes remained statistically significant after false discovery rate adjustment for each of these features.

This pattern was not uniform across all clinical features. Reported anemia was most prevalent in Class 1 (8.3%), compared with Classes 2 (6.1%) and 3 (5.1%). Swallowing difficulties, food regurgitation, and pain on swallowing did not differ significantly across classes. These findings indicate heterogeneous clinical feature profiles, with several common symptoms occurring more frequently in the two classes characterized by higher psychosocial burden.

### 3.6. Endoscopic Findings Across Latent Classes

**Figure 2:**
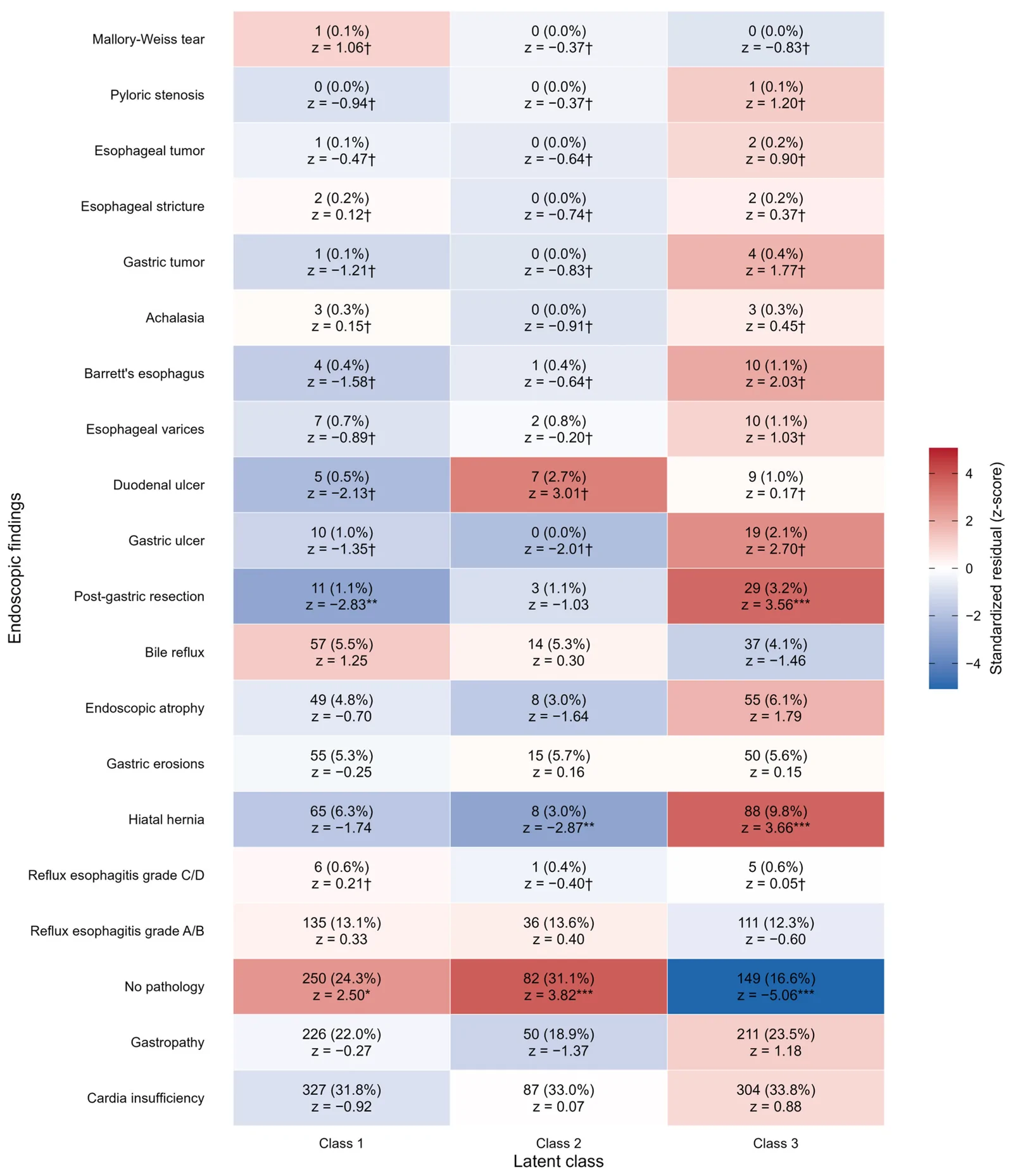
Endoscopic findings across latent classes, presented as patient counts, within-class percentages, and standardized residuals. Each cell shows the number and within-class percentage of patients with the endoscopic finding, followed by its standardized residual (z). Red indicates more patients with the finding than expected under independence, and blue indicates fewer. One, two, and three asterisks denote exploratory thresholds of |z| > 1.96, > 2.58, and > 3.29, respectively. A dagger (†) identifies findings with sparse expected cell counts; their residuals are descriptive, and asterisks are omitted.

Endoscopic findings differed significantly across the identified latent classes, with distinct patterns observed in both relative distribution and absolute prevalence (Figure 2).

The proportion of endoscopies without visible pathology was highest in Class 2 (31.1%), followed by Class 1 (24.3%) and Class 3 (16.6%). Conversely, Class 3 had the highest prevalence of hiatal hernia (9.8%, compared with 6.3% in Class 1 and 3.0% in Class 2) and post-gastric resection findings (3.2%, compared with 1.1% in both other classes). Standardized residuals indicated relative overrepresentation of these structural findings and underrepresentation of examinations without pathology in Class 3.

Grade A/B reflux esophagitis showed similar observed prevalences across Classes 1, 2, and 3 (13.1%, 13.6%, and 12.3%, respectively), as did gastric erosions (5.3%, 5.7%, and 5.6%). Gastric ulcer was most frequent in Class 3 (2.1%), whereas duodenal ulcer was most frequent in Class 2 (2.7%). These ulcer findings and several other less frequent abnormalities had sparse expected cell counts; their class-specific residuals should therefore be interpreted descriptively.

Taken together with the clinical feature profiles, these findings illustrate symptom–pathology discordance: Class 2 had the highest prevalence of several common symptoms but also the largest proportion of examinations without visible pathology, whereas Class 3 had fewer reported symptoms alongside a higher prevalence of selected structural findings.

### 3.7. Histological Findings Across Latent Classes

**Figure 3:**
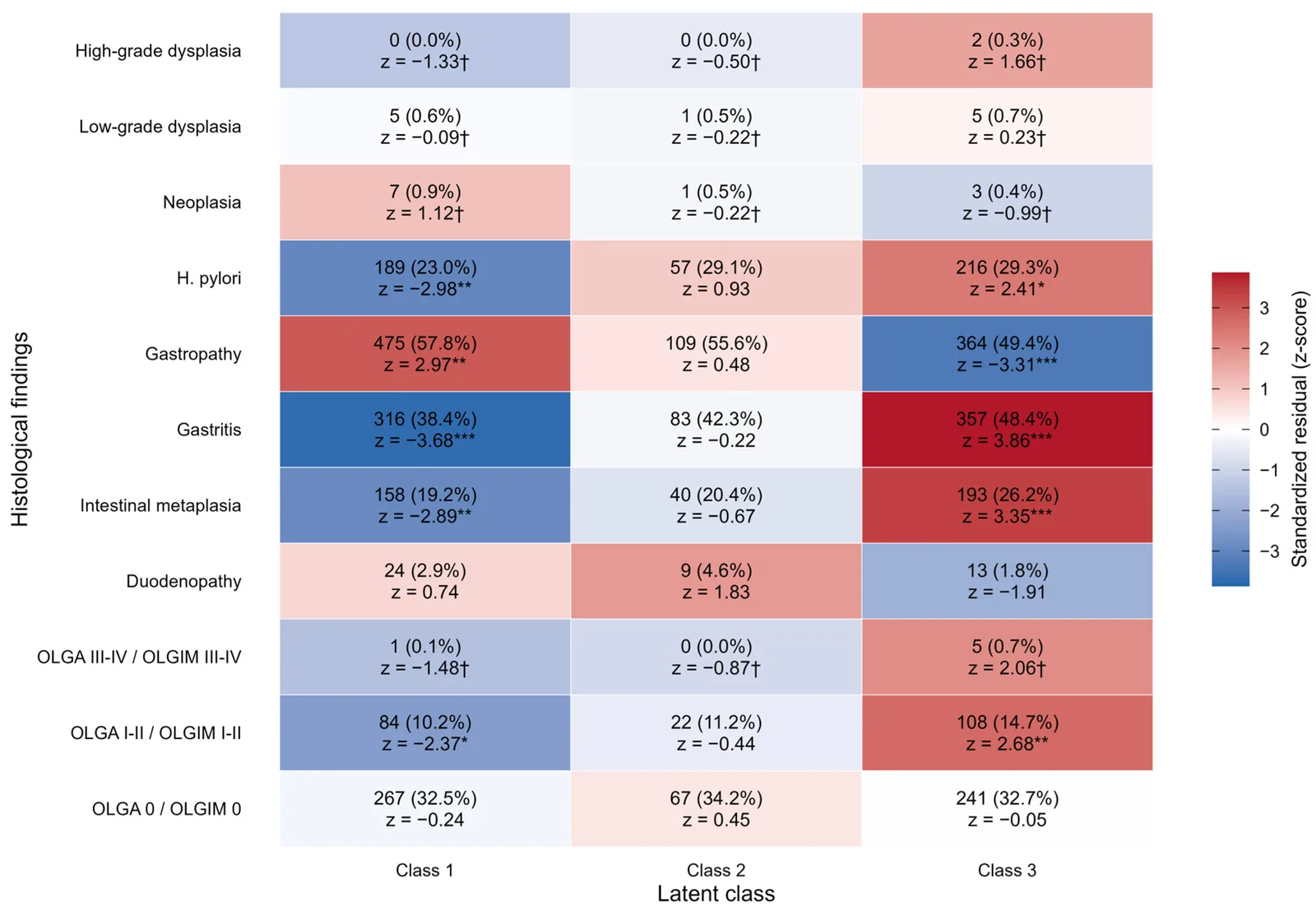
Histological findings across latent classes among patients with histological data, presented as patient counts, within-class percentages, and standardized residuals. Analyses were restricted to patients with histology. Each cell shows the number and within-class percentage of patients with the histological finding, followed by its standardized residual (z). Red indicates more patients with the finding than expected under independence, and blue indicates fewer. One, two, and three asterisks denote exploratory thresholds of |z| > 1.96, > 2.58, and > 3.29, respectively. A dagger (†) identifies findings with sparse expected cell counts; their residuals are descriptive, and asterisks are omitted. OLGA, Operative Link on Gastritis Assessment; OLGIM, Operative Link on Gastric Intestinal Metaplasia Assessment.

Histological findings differed across latent classes, with distinct patterns observed in the relative distribution of inflammatory, premalignant, and non-specific mucosal changes (Figure 3).

Among patients with histological data, Class 3 had the highest observed prevalence of gastritis (48.4%) and intestinal metaplasia (26.2%), compared with 38.4% and 19.2% in Class 1 and 42.3% and 20.4% in Class 2, respectively. *H. pylori* infection was identified in 29.3% of patients with histological data in Class 3, 29.1% in Class 2, and 23.0% in Class 1. Conversely, gastropathy was most prevalent in Class 1 (57.8%), followed by Class 2 (55.6%) and Class 3 (49.4%). Overall class differences remained statistically significant after false discovery rate adjustment for these four findings.

Combined OLGA/OLGIM stages I–II were most frequent in Class 3 (14.7%), but the overall difference did not remain statistically significant after false discovery rate correction (FDR-adjusted *p* = 0.056). Advanced stages III–IV were recorded in six patients: one in Class 1 and five in Class 3. Their distribution did not differ significantly across classes.

Histologically confirmed neoplasia was identified in 11 patients, comprising six neuroendocrine tumors, four carcinoma diagnoses, and one melanoma metastasis. Low-grade and high-grade dysplasia were identified in 11 and two patients, respectively. No statistically significant class differences were detected for these findings; the small numbers limit interpretation of their distributions. Overall, the histological profiles showed higher prevalences of selected inflammatory and premalignant findings in Class 3 despite its lower psychosocial burden and lower prevalence of several common symptoms.

Additional analyses examined associations between latent class membership and the individual findings before and after adjustment for age and sex (Appendix A, Table A1). Of the 12 clinical features associated with class membership before adjustment after false discovery rate correction, 11 remained significant after adjustment; reported anemia no longer met this threshold. Four endoscopic and four histological findings were significant before adjustment, whereas none remained significant after both age and sex adjustment and false discovery rate correction. After adjustment for age and sex, associations with absence of visible endoscopic pathology, duodenal ulcer, gastritis, gastropathy, and *H. pylori* infection had uncorrected *p*-values below 0.05, but none remained statistically significant after false discovery rate correction.

### 3.8. Clinical Feature-Pathology Concordance and Discordance Across Latent Classes

Clinical feature-pathology concordance profiles differed across latent classes within the histology subgroup (χ^2^ = 55.71; *p* < 0.001; Cramér’s V = 0.126). As shown in Table 10, concordant low findings were the most frequent profile in every class, accounting for 54.5% of Class 1, 44.4% of Class 2, and 54.5% of Class 3. Clinical feature-dominant discordance was most frequent in Class 2 (25.5%), compared with Class 1 (18.7%) and Class 3 (10.9%). Conversely, pathology-dominant discordance was most frequent in Class 3 (29.7%), compared with Class 2 (20.4%) and Class 1 (19.3%). Concordant high findings were uncommon, ranging from 4.9% to 9.7%. Together, these findings provide an integrative summary of the main study pattern, showing that latent classes differed not only in clinical feature and pathology profiles separately, but also in the degree and direction of clinical feature-pathology mismatch.

### 3.9. ROC Curves for Endoscopic and Histological Yield

**Figure A1:**
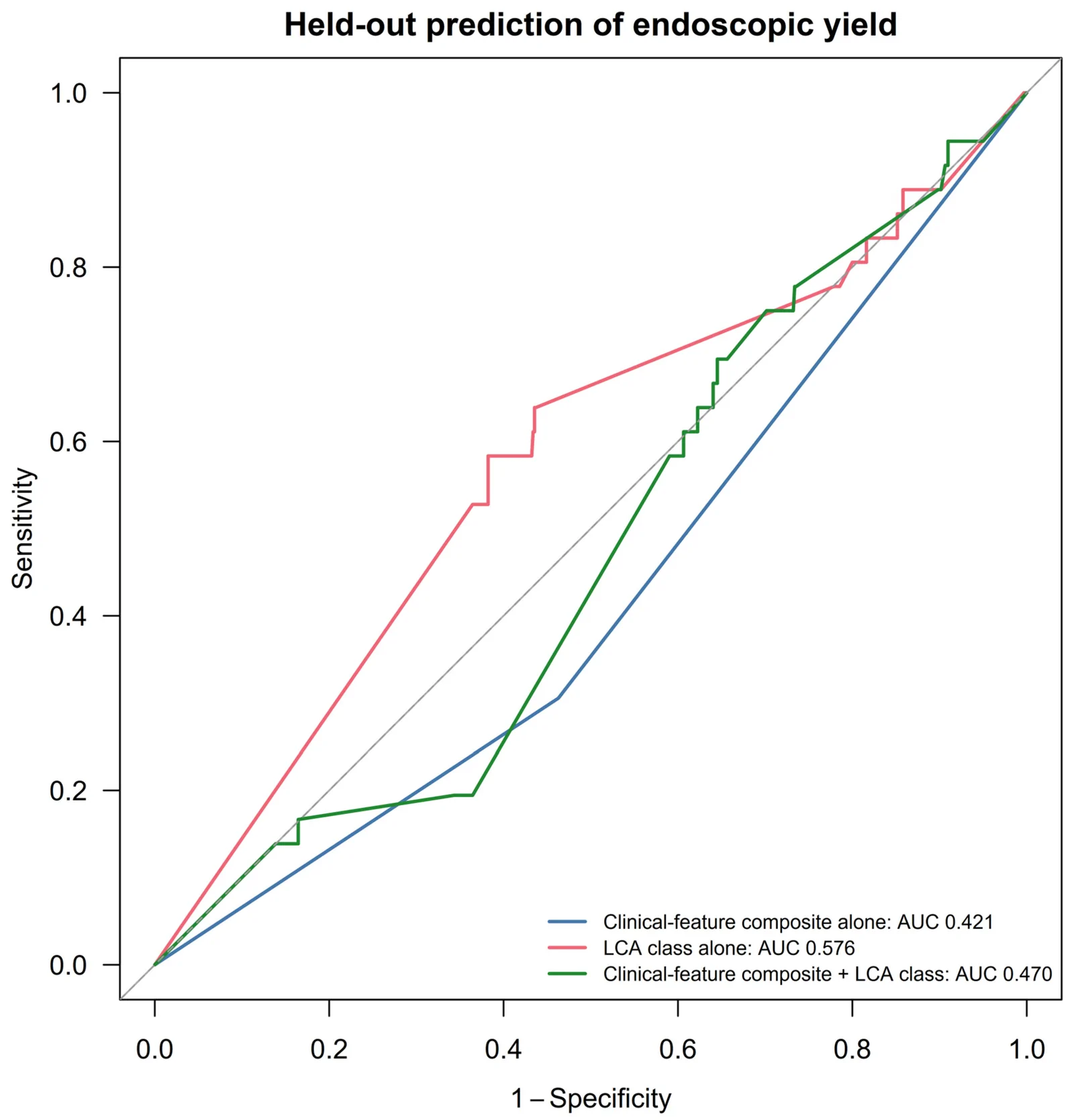
Receiver operating characteristic curves for prediction of endoscopic yield using the clinical feature composite, latent class membership, and their combination. Curves show the performance of three logistic regression models evaluated in the same held-out test sample: the clinical feature composite alone (blue; AUC 0.421), latent class membership alone (pink; AUC 0.576), and their combination (green; AUC 0.470). The grey diagonal line represents chance-level discrimination. AUC, area under the receiver operating characteristic curve; LCA, latent class analysis.

**Figure A2:**
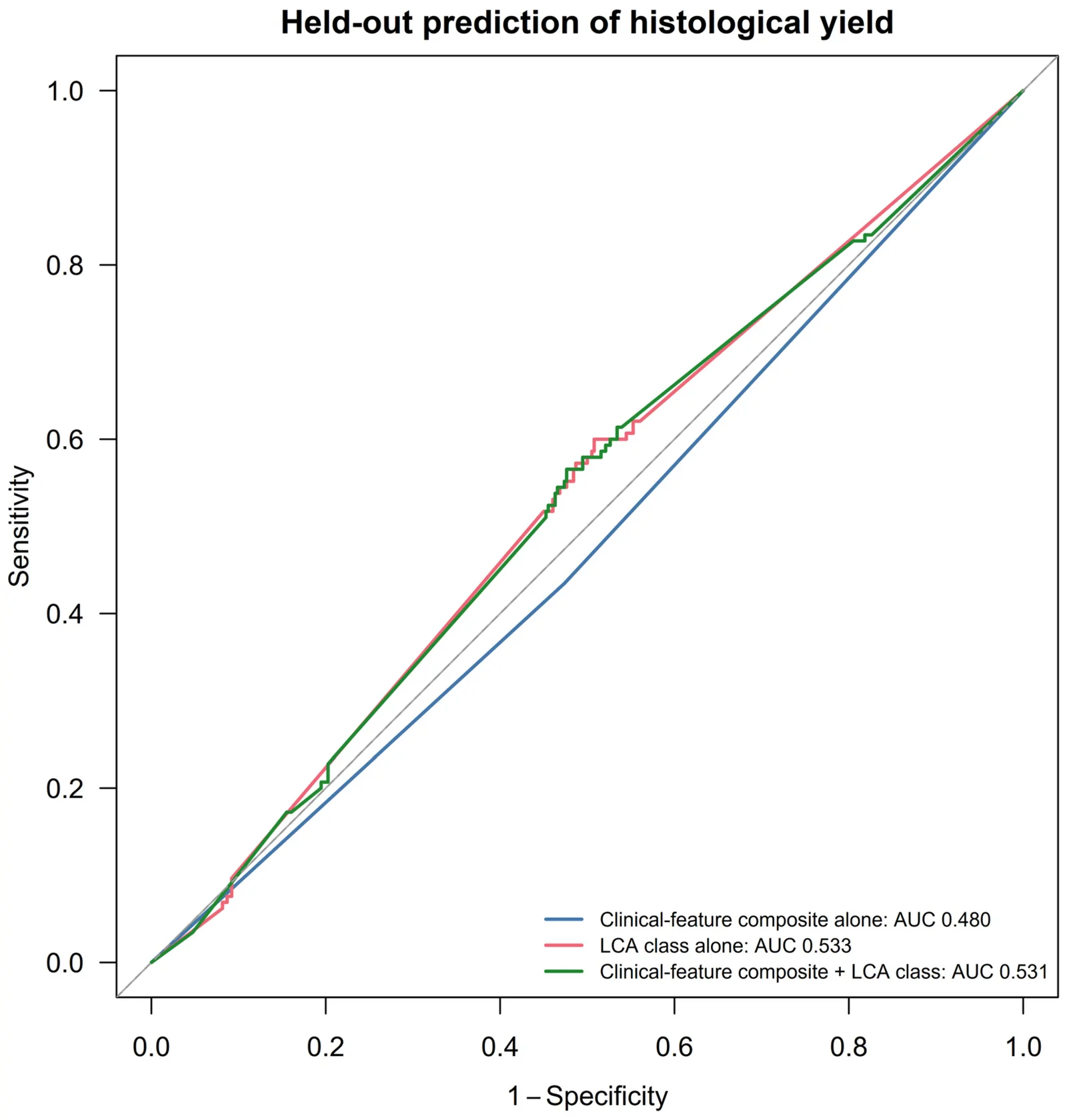
Receiver operating characteristic curves for prediction of histological yield using the clinical feature composite, latent class membership, and their combination. Curves show the performance of three logistic regression models evaluated in the same held-out test sample of patients with available histology: the clinical feature composite alone (blue; AUC 0.480), latent class membership alone (pink; AUC 0.533), and their combination (green; AUC 0.531). The grey diagonal line represents chance-level discrimination. AUC, area under the receiver operating characteristic curve; LCA, latent class analysis.

Discrimination was evaluated in held-out test samples using models containing the clinical feature composite, latent class membership, or both. For endoscopic yield, AUC values were 0.421 (95% CI 0.343–0.500) for the clinical feature composite alone, 0.576 (95% CI 0.478–0.675) for latent class membership alone, and 0.470 (95% CI 0.380–0.560) for their combination (Appendix A, Figure A1). For histological yield, the corresponding AUC values were 0.480 (95% CI 0.433–0.528), 0.533 (95% CI 0.482–0.584), and 0.531 (95% CI 0.477–0.585) (Appendix A, Figure A2). Models incorporating latent class membership showed a modest numerical gain in discrimination compared with the clinical feature composite alone for both outcomes. Although these differences were not statistically significant after Holm correction and overall discrimination remained limited, they suggest a potential contribution of lifestyle-based phenotyping that warrants further investigation.

## 4. Discussion

### 4.1. Principal Findings

In this large real-world cohort of adults undergoing upper gastrointestinal endoscopy, latent class analysis identified three clinically interpretable phenotypes based on lifestyle, behavioral, and psychosocial characteristics. Classes 1 and 2 exhibited higher psychosocial burden, with comparatively low substance exposure in Class 1 and greater substance exposure in Class 2; Class 3 exhibited lower psychosocial burden. Several common symptoms were most prevalent in Class 2 and least prevalent in Class 3. Conversely, Class 3 showed higher prevalences of selected structural endoscopic findings, gastritis, and intestinal metaplasia, together with the highest proportion of pathology-dominant discordance. These findings illustrate differing patterns of clinical feature-pathology mismatch across the identified phenotypes.

This discordance was supported by multiple complementary analyses. The clinical feature composite demonstrated limited diagnostic performance, with low positive predictive values for clinically meaningful endoscopic and histological pathology. In addition, correlations between clinical feature burden and pathology burden were negligible, indicating that increasing clinical feature burden did not correspond to greater objective disease burden. Concordance analyses further showed that both clinical feature-dominant and pathology-dominant discordance occurred, with pathology-dominant discordance being more frequent. Together, these findings indicate that the selected clinical feature measures provided limited information about the presence and number of the endoscopic and histological findings evaluated in this cohort.

Clinically, the observed differences highlight the limitations of using symptom burden alone to infer structural or histological disease. Psychosocial factors may contribute to symptom reporting, but our study did not assess physiological mechanisms or establish disorders of gut–brain interaction. Conversely, a limited symptom burden does not exclude clinically relevant pathology. These findings are descriptive and do not support changes to guideline-based indications for endoscopy without prospective validation.

### 4.2. Symptom-Pathology Dissociation: Possible Explanations

Previous studies have established associations between psychosocial factors and gastrointestinal symptom experience, including in disorders of gut–brain interaction [7,8,18,19]. However, the present study did not directly assess visceral sensitivity, autonomic function, motility, or diagnostic criteria for functional gastrointestinal disorders. Therefore, the observed associations between psychosocial burden and symptom profiles cannot be attributed to a specific gut–brain mechanism.

In the present cohort, Classes 1 and 2 were characterized by higher perceived stress, increased stress, and anxiety than Class 3. Several common gastrointestinal and systemic symptoms were most prevalent in Class 2, followed by Class 1, and least prevalent in Class 3. Class 2 also had the highest proportion of endoscopies without visible pathology, whereas Class 3 had higher prevalences of selected structural findings, gastritis, and intestinal metaplasia. These patterns are consistent with a possible contribution of psychosocial factors to symptom experience, although the observational design does not establish the mechanisms underlying these associations.

Several possible explanations may contribute to the observed symptom-pathology differences, including psychosocial context, symptom perception, medication effects, disorders of gut–brain interaction, and conditions that are not visible on routine endoscopy. A normal or low-yield endoscopy does not by itself confirm a functional disorder or exclude non-erosive reflux disease. The present analyses cannot distinguish between these explanations or establish causality; clinical assessment and further testing should be guided by individual indications.

### 4.3. Class-Specific Interpretation

Class 1 represented a phenotype of high psychosocial burden with comparatively low substance exposure. High perceived stress and increased stress were common despite predominantly never alcohol use and no nicotine use in the preceding 12 months. Several common clinical features were more prevalent than in Class 3, although their prevalences were generally lower than in Class 2. This profile illustrates that substantial psychosocial burden and frequent symptoms were not confined to patients with greater substance exposure.

Class 2 combined greater substance exposure with high psychosocial burden. Frequent nicotine use and recreational drug use were most prevalent in this class, while former alcohol users constituted the largest alcohol category. Class 2 also had the highest anxiety prevalence and the highest prevalence of several common symptoms, alongside the largest proportion of endoscopies without visible pathology and clinical feature-dominant discordance. Lifestyle exposures, including alcohol use, smoking, obesity, and physical inactivity, have been associated with gastrointestinal disease risk [12,13,14]. Nevertheless, the observed Class 2 profile did not show uniform enrichment of objective pathology, illustrating that the coexistence of these exposures and psychosocial burden does not necessarily correspond to a greater prevalence of the endoscopic and histological findings assessed.

Class 3 represented a lower psychosocial burden phenotype, with fewer patients with short sleep duration, less prolonged sitting, and lower sleep medication use. Nevertheless, overweight and obesity combined were most prevalent in this class. Several common symptoms were less frequent, while selected structural endoscopic findings, gastritis, and intestinal metaplasia were more prevalent. Class 3 also had the highest proportion of pathology-dominant discordance. This profile illustrates that lower psychosocial burden and fewer reported symptoms did not uniformly coincide with fewer objective abnormalities.

Together, these phenotypes show that patients referred for upper gastrointestinal endoscopy are clinically heterogeneous. Symptom burden, psychosocial characteristics, lifestyle exposures, and objective pathology combine in different ways, which may partly explain why conventional symptom-based approaches have limited ability to predict endoscopic or histological findings.

### 4.4. Implications for Endoscopy Triage

The limited discriminatory performance of the clinical feature composite is relevant to interpreting symptom-based assessment in patients referred for endoscopy. In the present cohort, the composite showed limited sensitivity, low specificity, and low positive predictive values for clinically meaningful pathology, while clinical feature burden was not meaningfully correlated with objective pathology burden. These findings apply to the selected composite and should not be interpreted as diminishing the clinical importance of individual alarm features or changing guideline-based indications for endoscopy.

This is consistent with large-scale evidence showing that the diagnostic yield of symptomatic gastroscopy is often limited. A British Society of Gastroenterology analysis of 382,370 diagnostic gastroscopies found that most symptomatic procedures showed normal findings or only minor pathology [9]. Similarly, a multicenter study of patients below 60 years found that although alarm features were associated with higher risk of major endoscopic findings than dyspepsia alone, the absolute prevalence of major findings and upper gastrointestinal cancer remained low [10]. Other studies have also reported weak or inconsistent associations between upper gastrointestinal symptoms and endoscopic outcomes [11].

Current dyspepsia guidelines recommend endoscopy particularly in older patients and selected higher-risk contexts, while recognizing that symptom presentation alone is an imperfect guide to organic pathology [3]. A more multidimensional risk assessment that incorporates not only symptoms, but also age, sex, *H. pylori* status, medication use, prior gastric pathology, lifestyle exposures, psychosocial burden, and local disease prevalence merits prospective evaluation. Such an approach may help reduce low-yield procedures among symptom-dominant patients while improving recognition of pathology-enriched patients with limited symptoms, although these potential benefits remain to be established through further investigation.

Alongside improving pre-endoscopy risk assessment, artificial intelligence (AI) and computer-assisted endoscopy offer opportunities to strengthen assessment during the procedure. Emerging applications include lesion detection and characterization, monitoring of mucosal inspection completeness, and clinical decision support [20]. These tools may help standardize endoscopic assessment and support recognition of subtle abnormalities. Nevertheless, improvements in examination quality do not necessarily translate into increased diagnostic yield: a recent multicenter randomized trial reduced blind spots but did not significantly improve gastric neoplasm detection after centralized pathological review [21]. AI-assisted endoscopy should therefore be considered a potential complement to clinical assessment, with benefits requiring validation for specific applications and settings. Our study did not evaluate these technologies, and their capacity to improve lesion detection should be distinguished from predicting pathology before endoscopy or explaining symptom-pathology discordance.

### 4.5. Value of Multidimensional Phenotyping

This study illustrates the value of multidimensional phenotyping for characterizing patient heterogeneity and examining symptom-pathology dissociation in upper gastrointestinal endoscopy. Traditional variable-centered analyses assess average associations between isolated predictors and outcomes, but may obscure clinically meaningful patient heterogeneity. In contrast, latent class analysis is a person-centered approach that identifies unobserved subgroups sharing similar patterns across multiple indicators [16,22]. In the present study, phenotypes were derived from lifestyle, behavioral, anthropometric, and psychosocial indicators. Clinical features, endoscopic findings, and histological findings were subsequently used to characterize these phenotypes and examine their clinical feature-pathology concordance patterns.

The exploratory ROC analyses provide additional context for interpreting the latent phenotypes. Models incorporating latent class membership showed numerically higher AUC values than the clinical feature composite alone for both endoscopic and histological yield, although the differences were not statistically significant after correction for multiple comparisons and overall discrimination remained limited. These findings support further investigation of lifestyle-based phenotyping while emphasizing its current role in characterizing symptom-pathology patterns. The identified classes should therefore be interpreted as descriptive phenotypes whose potential contribution to clinical decision-making requires further validation.

This approach may be relevant for precision gastroenterology by addressing the limited integration of clinical symptoms and modifiable lifestyle factors within a unified analytical framework. Considering these domains together may help characterize clinically meaningful patient subgroups and contextualize their endoscopic and histological findings alongside psychosocial and behavioral profiles. Such a framework could support more individualized post-endoscopy assessment, including consideration of histological risk, *H. pylori* management, surveillance needs, and integrated care addressing gut–brain interaction, stress, sleep, anxiety, and behavioral exposures. Its potential to complement guideline-based endoscopy selection and reduce low-yield procedures remains a question for prospective validation.

### 4.6. Strengths and Limitations

The main strengths of this study include the large real-world endoscopy cohort, integration of patient-reported symptoms with psychosocial, lifestyle, endoscopic, and histological data, and use of robust latent class methodology. Model selection considered statistical fit, class size, interpretability, and consistency across imputed datasets. Class assignments showed substantial agreement across the 20 imputations, with a mean pairwise adjusted Rand index of 0.863 ± 0.028. An additional strength is the explicit assessment of symptom-pathology concordance and discordance, which provides a clinically intuitive summary of the central finding.

Several limitations should be acknowledged.

First, the retrospective observational design precludes causal inference. Although clinical feature-dominant discordance was more frequent in the classes characterized by higher psychosocial burden, the direction of this relationship cannot be determined.

Second, the same single-center setting may limit external validity. Although the common institutional workflow and use of a single pathology laboratory enhanced internal consistency, these features do not eliminate center-specific referral, selection, or practice effects; endoscopic practices, pathology reporting, and local disease prevalence may differ across institutions and healthcare systems. The identified phenotypes and symptom-pathology associations should therefore not be assumed to generalize directly beyond this cohort and require external validation in independent gastroenterology centers, ideally through prospective multicenter studies. Differences in referral indications may also have influenced the observed symptom-pathology associations. Patients referred for symptom evaluation may differ from those referred for investigation of suspected disease or follow-up of previously diagnosed conditions. Although recorded referral diagnoses were summarized descriptively, the analyses did not account for referral diagnosis. Consequently, the observed associations may partly reflect differences in referral patterns and should be interpreted within this selected outpatient endoscopy cohort.

Age and sex differed across the latent classes and may partly account for the observed differences in endoscopic and histological findings. Although neither variable was included among the LCA indicators, their exclusion from class derivation does not eliminate potential confounding of subsequent class–finding associations. Additional adjustment analyses showed that most clinical-feature associations persisted, whereas no overall class associations with individual endoscopic or histological findings remained statistically significant after both demographic adjustment and false discovery rate correction (Appendix A, Table A1). Accordingly, the observed pathology profiles should be interpreted as descriptive differences within this cohort, without assuming that they are independent of its demographic composition. These results do not exclude underlying associations, particularly for uncommon findings with limited statistical precision.

Third, lifestyle and psychosocial variables were self-reported and may be affected by recall or reporting bias.

We did not measure autonomic nervous system function, visceral sensitivity, or esophageal acid exposure, nor did we assess validated diagnostic criteria for disorders of gut–brain interaction. Thus, the associations described here do not establish a functional gastrointestinal diagnosis or a specific neurogastroenterological mechanism. Negative or low-yield endoscopic findings may coexist with conditions requiring additional clinical assessment.

Fourth, histological data were available only for patients who underwent biopsy, and biopsy sampling was performed at the discretion of the endoscopist, introducing potential selection variability. Comparisons between patients with and without histology identified differences of limited magnitude in recreational drug use and BMI distribution among the behavioral, lifestyle, and psychosocial characteristics examined. Nevertheless, these comparisons cannot exclude selection related to clinical indications, endoscopic appearance, or other unmeasured factors. The histological distributions in Figure 3 therefore describe patients who underwent biopsy and should not be generalized to the entire endoscopy cohort.

An important additional limitation was the absence of reliably characterized proton pump inhibitor (PPI) exposure and previous *H. pylori* eradication therapy. Medication information was collected through patient-reported free-text fields without verification against medical records, creating potential under-reporting and exposure misclassification. Self-reported previous *H. pylori* infection did not establish whether eradication therapy had been administered or whether eradication was successful. Consequently, these exposures could not be incorporated reliably into the analyses. PPI therapy can promote healing of acid-related mucosal lesions and reduce the sensitivity of biopsy-based *H. pylori* detection, while previous eradication may reduce gastric inflammation. These treatment effects could alter the relationship between reported symptoms and findings at the index endoscopy. Because treatment histories were insufficiently characterized, we could not determine their distribution across classes or quantify their influence on the observed associations. Residual confounding and treatment-related differences in pathology detection therefore remain important alternative explanations for part of the observed symptom-pathology dissociation.

Fifth, the latent classes were derived from available questionnaire variables and should not be interpreted as definitive biological entities without external validation.

Finally, data on symptom duration, family history, diet, socioeconomic factors, and longitudinal outcomes were limited or unavailable.

### 4.7. Future Directions

Future studies should validate these phenotypes in independent multicenter cohorts and evaluate whether class membership improves prediction of high-yield endoscopic or histological findings beyond standard clinical variables. Prospective research should also assess whether these phenotypes predict symptom persistence, repeat healthcare use, treatment response, quality of life, and development of premalignant or malignant pathology. Ultimately, phenotype-guided approaches may support more precise triage and more tailored post-endoscopy management.

## 5. Conclusions

This study identified three clinically distinct phenotypes among patients undergoing upper gastrointestinal endoscopy and demonstrated a substantial dissociation between patient-reported symptom burden and objectively detected structural and histological pathology. Class 1 combined high psychosocial burden with comparatively low substance exposure and intermediate prevalences of several common symptoms. Class 2 exhibited greater substance exposure, the highest prevalences of these symptoms, and the largest proportion of endoscopies without visible pathology. Class 3 had lower psychosocial burden and fewer reported symptoms alongside higher prevalences of selected structural and histological findings. In parallel, the clinical feature composite showed limited diagnostic performance, and increasing clinical feature burden was not associated with greater objective pathology burden. These findings indicate that the presence and number of selected clinical features were limited proxies for the objective pathology assessed and highlight the heterogeneity of patients referred for endoscopy. Incorporating psychosocial, lifestyle, clinical, endoscopic, and histological information may provide a more informative framework for patient characterization and future risk stratification than symptom-based assessment alone. Given the single-center design, these findings should be externally validated in independent and multicenter gastroenterology populations before broader clinical application.

## Figures and Tables

**Table 1:** Operational definitions and categorization of variables included in latent class analysis.

Variable	Category
Current nicotine use	None in the last 12 months
	Occasional use: monthly or less, or 2–4 times/month
	Frequent use: 2–4 times/week, 4–6 times/week, or daily
Alcohol exposure	Never use: no reported lifetime alcohol use
	Former use: reported lifetime alcohol use, but no current consumption
	Current low consumption: current consumption less than once/week or 1–4 times/week, with <1 drink or 1–2 drinks per occasion
	Current moderate-to-high consumption: current consumption of 3–4 or ≥5 drinks per occasion at frequencies below once/week or 1–4 times/week; or consumption 5–6 times/week or daily with a recorded quantity per occasion
Recreational drug use	No
	Yes, based on either lifetime or current drug use
Sedentary behavior	≤4 h/day
	>4 and ≤8 h/day
	>8 h/day
Regular physical activity	No
	Yes
Sleep duration	≤6 h/night
	>6 and ≤8 h/night
	>8 h/night
Sleep medication use	No
	Yes
Perceived stress level	Low: score ≤ 3
	Moderate: score 4–6
	High: score ≥ 7
Increased stress	No
	Yes
Anxiety	No
	Yes
Occupational risk exposure	No
	Yes
Body mass index category	Underweight: <18.5 kg/m^2^
	Normal weight: 18.5 to <25 kg/m^2^
	Overweight: 25 to <30 kg/m^2^
	Obesity: ≥30 kg/m^2^

**Table 2:** Baseline characteristics of the study population.

Characteristic	Total Sample (n = 2192)
Age, years, mean ± SD	50.8 ± 14.9
Female sex, n (%)	1455 (66.4)
BMI, kg/m^2^	26.6 ± 5.3
Underweight, n (%)	65 (3.0)
Normal weight, n (%)	881 (40.2)
Overweight, n (%)	725 (33.1)
Obesity, n (%)	519 (23.7)
Higher education, n (%)	1284 (59.5)
Employed, n (%)	1354 (62.8)

Note. Values are presented as mean ± standard deviation or n (%). SD, standard deviation; BMI, body mass index.

**Table 3:** Distribution of recorded referral diagnoses among patients undergoing outpatient upper gastrointestinal endoscopy.

Referral Diagnosis Category	ICD-10 Codes	N (%)
Gastritis and duodenitis	K29, K29.0, K29.1, K29.3–K29.9	567 (25.9)
Gastroesophageal reflux disease and esophagitis	K20, K21, K21.0, K21.9	541 (24.7)
Observation for suspected malignancy	Z03.1, Z03.115, Z03.116, Z03.122	481 (21.9)
Observation for other suspected or unspecified conditions	Z03, Z03.00, Z03.8, Z03.9	97 (4.4)
Peptic ulcer disease	K25, K25.0, K25.3, K25.7, K25.9; K26, K26.3, K26.7, K26.9; K27, K27.9; K28, K28.1	84 (3.8)
Malignant neoplasms of digestive organs	C15, C15.9; C16, C16.2, C16.8, C16.9; C17.0, C17.9; C18, C18.0, C18.2, C18.4, C18.7, C18.9; C19, C20, C21.0, C22, C22.0	75 (3.4)
Functional dyspepsia	K30	60 (2.7)
Intestinal disorders and fecal abnormalities	K50; K51, K51.0, K51.2, K51.9; K52, K52.3; K57; K58, K58.3; K59, K59.0, K59.9; K63.9; R19.5	55 (2.5)
Anemia or iron deficiency	D50, D50.8, D50.9, D51, D64, E61.1	47 (2.1)
Malignant neoplasms at other sites and hematological malignancies	C01, C09, C32, C43; C50, C50.1, C50.4, C50.5, C50.9; C53, C61, C64, C82.1, C83.7, C90.0	34 (1.6)
Abdominal pain, heartburn, dysphagia, or abnormal weight loss	R10, R10.1, R10.3, R10.4, R12, R13, R63.4	30 (1.4)
Other gastric and duodenal disorders	K31, K31.7, K31.8, K31.9	25 (1.1)
Follow-up, screening, history, and other health-service encounter codes	Z08.0, Z09, Z12.0, Z76.8, Z80, Z85.0, Z90.3, Z94.0, Z94.4	17 (0.8)
Other esophageal disorders, including esophageal varices	I85, I85.9; K22, K22.0, K22.2, K22.4, K22.7, K22.9	15 (0.7)
Hepatobiliary and pancreatic disorders	K74, K74.3, K74.5, K76.6, K76.8, K80, K83.8, K85, K86.8	13 (0.6)
Celiac disease	K90.0	11 (0.5)
Unspecified gastrointestinal bleeding	K92.2	11 (0.5)
Benign, uncertain-behavior, or in situ neoplasms of the upper gastrointestinal tract	D00.2, D13.0, D13.1, D13.13, D37.1	9 (0.4)
Diaphragmatic hernia	K44, K44.9	5 (0.2)
Other referral diagnoses	B37, B98.0, E03.8, E66, G35, I15, K92, K92.9, L40.5, N18.5, R89	15 (0.7)

Note. Data are presented as n (%), with percentages calculated using the total cohort of 2192 patients. One ICD-10 referral diagnosis was recorded per patient, and diagnoses were grouped into mutually exclusive categories. Referral diagnoses were obtained from the referring physicians’ referrals and do not necessarily represent conditions confirmed by endoscopy or histology.

**Table 4:** Diagnostic performance of the clinical feature composite for endoscopic yield, histological yield, and high-yield gastroscopy.

Parameter	Endoscopic Yield	Histological Yield	High-Yield Gastroscopy
N	1755	1755	1755
True positive	41	216	235
True negative	927	701	672
False positive	743	568	549
False negative	44	270	299
Sensitivity (95% CI)	0.48 (0.37–0.59)	0.44 (0.40–0.49)	0.44 (0.40–0.48)
Specificity (95% CI)	0.56 (0.53–0.58)	0.55 (0.52–0.58)	0.55 (0.52–0.58)
PPV (95% CI)	0.05 (0.04–0.07)	0.28 (0.24–0.31)	0.30 (0.27–0.33)
NPV (95% CI)	0.96 (0.94–0.97)	0.72 (0.69–0.75)	0.69 (0.66–0.72)

Note. All analyses in this table were restricted to the 1755 patients with available histological data to permit comparison of the three yield outcomes within the same sample. A positive clinical feature composite, defined as the presence of at least one selected clinical feature, was used as the index test. CI, confidence intervals; PPV, positive predictive value; NPV, negative predictive value.

**Table 5:** Spearman correlations between clinical feature burden and pathology burden.

Comparison	Spearman’s ρ	*p*-Value
Clinical feature burden vs. endoscopic burden	0.026	0.225
Clinical feature burden vs. histological burden	−0.012	0.626
Clinical feature burden vs. combined pathology burden	−0.006	0.788

Note. Clinical feature burden counts the nine selected features included in the clinical feature composite. Endoscopic and histological burdens count the findings included in their respective yield definitions; combined pathology burden is their sum. Associations were assessed using Spearman’s rank correlation coefficient (ρ).

**Table 6:** Concordance and discordance between clinical feature burden and objective pathology.

Clinical Feature Burden	Objective Pathology	Group	N (%)
High	Present	Concordant high: high clinical feature burden + pathology	116 (6.6)
Low	Absent	Concordant low: low clinical feature burden + no pathology	937 (53.4)
High	Absent	Clinical feature-dominant discordance	284 (16.2)
Low	Present	Pathology-dominant discordance	418 (23.8)

Note. Clinical feature burden was calculated as the total number of selected clinical features reported by each patient. Low clinical feature burden was defined as 0–1 reported features, and high clinical feature burden as ≥2 reported features. Objective pathology was defined as high-yield gastroscopy, indicating clinically significant endoscopic and/or histological pathology. Percentages are calculated from the total number of patients included in the concordance analysis.

**Table 7:** Model fit statistics for latent class solutions.

Number of Classes	Mean BIC ± SD	Mean Entropy ± SD	Mean Smallest Class, %
1	38,031.5 ± 42.9	—	100.0
2	37,264.9 ± 37.4	0.641 ± 0.006	44.0
3	37,237.2 ± 39.1	0.655 ± 0.005	13.4
4	37,278.8 ± 37.4	0.611 ± 0.010	11.7
5	37,353.5 ± 37.9	0.635 ± 0.009	7.4
6	37,444.0 ± 39.2	0.637 ± 0.027	6.5

Note. Values are mean ± SD across 20 multiply imputed datasets. BIC, Bayesian Information Criterion; SD, standard deviation.

**Table 10:** Distribution of clinical feature-pathology concordance and discordance profiles across latent classes.

Latent Class	N	Concordant Low, n (%)	Pathology-Dominant Discordance, n (%)	Clinical Feature-Dominant Discordance, n (%)	Concordant High, n (%)
Class 1	822	448 (54.5)	159 (19.3)	154 (18.7)	61 (7.4)
Class 2	196	87 (44.4)	40 (20.4)	50 (25.5)	19 (9.7)
Class 3	737	402 (54.5)	219 (29.7)	80 (10.9)	36 (4.9)

Note. Analysis included 1755 patients with available histology. Values are presented as number (percentage), with percentages calculated within each latent class. Objective pathology was defined as high-yield gastroscopy. Concordant and discordant profiles were defined according to clinical feature burden and objective pathology status. Concordant low denotes low feature burden with a negative pathology composite; concordant high denotes high feature burden with a positive composite. Pathology-dominant discordance denotes low feature burden with a positive composite, and clinical feature-dominant discordance denotes high feature burden with a negative composite. The overall distribution of profiles differed across classes (Pearson’s χ^2^ = 55.71, df = 6, *p* < 0.001; Cramér’s V = 0.126).

## Data Availability

The data supporting the findings of this study are not publicly available due to ethical and privacy restrictions related to patient-level clinical data. De-identified data may be made available from the corresponding author upon reasonable request and subject to approval by the relevant institutional and ethical authorities.

## Abbreviations

The following abbreviations are used in this manuscript:

AI	Artificial intelligence
AUC	Area under the receiver operating characteristic curve
BIC	Bayesian Information Criterion
BMI	Body mass index
CI	Confidence interval
FDR	False discovery rate
ICD-10	International Classification of Diseases, 10th Revision
LCA	Latent class analysis
NPV	Negative predictive value
OLGA	Operative Link on Gastritis Assessment
OLGIM	Operative Link on Gastric Intestinal Metaplasia Assessment
PPI	Proton pump inhibitor
PPV	Positive predictive value
ROC	Receiver operating characteristic
SD	Standard deviation
UGI	Upper gastrointestinal

## Appendix A

**Table A1:** Overall associations of latent class membership with clinical features, endoscopic findings, and histological findings before and after adjustment for age and sex.

Finding	Available N	Positive Cases, n	Unadjusted *p*	Unadjusted q	Adjusted *p*	Age- and Sex-Adjusted q
Clinical features						
Swallowing difficulties	2192	62	0.595	0.595	0.507	0.507
Food regurgitation	2192	90	0.252	0.269	0.261	0.298
Pain on swallowing	2192	77	0.242	0.269	0.335	0.357
Vomiting	2192	33	<0.001	<0.001	0.005	0.010
Reported anemia	2192	147	0.024	0.032	0.114	0.152
Hematemesis	2192	5	0.166	0.205	0.141	0.173
Belching	2192	370	0.002	0.004	0.005	0.010
Heartburn	2192	485	0.006	0.010	0.020	0.029
Unexplained weight loss	2192	142	0.019	0.028	<0.001	<0.001
Epigastric burning	2192	369	0.004	0.006	0.008	0.015
Appetite changes	2192	196	<0.001	<0.001	<0.001	<0.001
Nausea	2192	248	<0.001	<0.001	0.012	0.020
Excessive sweating	2192	200	<0.001	<0.001	<0.001	<0.001
Abdominal pain	2192	566	<0.001	<0.001	<0.001	<0.001
Abdominal bloating	2192	788	<0.001	<0.001	<0.001	<0.001
Fatigue	2192	495	<0.001	<0.001	<0.001	<0.001
Endoscopic Findings						
Cardia insufficiency	2192	718	0.634	0.993	0.563	0.943
Gastropathy	2192	487	0.301	0.753	0.896	0.943
No pathology	2192	481	<0.001	<0.001	0.029	0.292
Reflux esophagitis A/B	2192	282	0.800	0.993	0.282	0.943
Reflux esophagitis C/D	2192	12	0.993	0.993	0.649	0.943
Hiatal hernia	2192	161	<0.001	0.005	0.153	0.813
Gastric erosions	2192	120	0.950	0.993	0.773	0.943
Endoscopic atrophy	2192	112	0.130	0.432	0.853	0.943
Bile reflux	2192	108	0.339	0.754	0.869	0.943
Post-gastric resection	2192	43	0.004	0.027	0.325	0.943
Gastric ulcer	2192	29	0.043	0.173	0.163	0.813
Duodenal ulcer	2192	21	0.009	0.047	0.014	0.281
Esophageal varices	2192	19	0.612	0.993	0.831	0.943
Barrett’s esophagus	2192	15	0.191	0.545	0.440	0.943
Achalasia	2192	6	0.890	0.993	0.852	0.943
Gastric tumor	2192	5	0.383	0.766	0.699	0.943
Esophageal stricture	2192	4	0.966	0.993	0.979	0.979
Esophageal tumor	2192	3	0.818	0.993	0.755	0.943
Pyloric stenosis	2192	1	0.724	0.993	0.404	0.943
Mallory-Weiss tear	2192	1	0.799	0.993	0.657	0.943
Histological Findings						
OLGA 0/OLGIM 0	1755	575	0.893	0.980	0.739	0.912
OLGA I-II/OLGIM I-II	1755	214	0.028	0.061	0.150	0.329
OLGA III-IV/OLGIM III-IV	1755	6	0.269	0.423	0.411	0.712
Duodenopathy	1755	46	0.065	0.120	0.975	0.975
Intestinal metaplasia	1755	391	0.004	0.014	0.133	0.329
Gastritis	1755	756	<0.001	0.004	0.011	0.116
Gastropathy	1755	948	0.004	0.014	0.039	0.142
*H. pylori*	1755	462	0.012	0.033	0.029	0.142
Neoplasia	1755	11	0.599	0.732	0.453	0.712
Low-grade dysplasia	1755	11	0.980	0.980	0.923	0.975
High-grade dysplasia	1755	2	0.540	0.732	0.746	0.912

Note. Each finding was analyzed as a binary outcome using mean bias-reduced logistic regression. Unadjusted models included latent class membership as a categorical predictor; adjusted models additionally included continuous age and sex. Reported *p*-values are overall two-degree-of-freedom Wald tests of class membership. Values labelled q are Benjamini–Hochberg FDR-adjusted *p*-values, calculated separately within each domain and separately for unadjusted and adjusted models. Histological analyses were restricted to patients with available histology. FDR, false discovery rate.
